# A Simple and Safe Approach for Maxillary Sinus Augmentation with the Advanced Surgical Guide

**DOI:** 10.3390/ijerph17113785

**Published:** 2020-05-27

**Authors:** Seoung-Won Cho, Byoung-Eun Yang, Kyeong-Jun Cheon, Won-Seok Jang, Ju-Won Kim, Soo-Hwan Byun

**Affiliations:** 1 Department of Oral and Maxillofacial Surgery, Hallym University College of Medicine, Anyang 14068, Korea; kotneicho@gmail.com (S.-W.C.); face@hallym.ac.kr (B.-E.Y.); shgusbabo@naver.com (K.-J.C.); wionnk@naver.com (W.-S.J.); kjw9199@hanmail.net (J.-W.K.); 2 Graduate School of Clinical Dentistry, Hallym University, Chuncheon 24252, Korea; 3 Institute of Clinical Dentistry, Hallym University, Chuncheon 24252, Korea

**Keywords:** implant, digital guide, navigation, sinus augmentation, lateral window technique

## Abstract

Objective: The development of digital dentistry has contributed to the astonishing advancement of implant dentistry. Furthermore, digital technology is expected to be applied extensively to sinus augmentation, which is an advanced technique for implant surgery. This study introduces a simple method for a safer and more precise lateral window opening for sinus augmentation using a navigation program. Methods: Five eligible patients with residual alveolar bone of 4 mm or less are presented, requiring lateral approach for sinus augmentation. Navigation system was opted for the sinus lift with simultaneous implant placement. Virtual planning started with establishing the adequate position of the lateral window based on the radiographic images and the scanned file of the dentition. The position of the window was indicated on the guide within the program. Afterwards, the virtually designed surgical guide was fabricated either with 3D printer or milling machine for the actual surgery. Results: All the patients who underwent surgery with the surgical guide showed no sign of clinical complications including pain, swelling, nausea, epistaxis, or early loss of the implants. Results of radiographic evaluation also showed adequate placement of the implants in a pre-planned position, and the sinus window was also formed in the desired location. Conclusion: Lateral window opening combined with digital navigation system is believed to be a promising technique for a more precise as well as safer sinus augmentation.

## 1. Introduction

The successful placement of an implant can be compromised in the posterior region of maxilla, primarily due to the lack of vertical dimension in alveolar bone [1]. The deficiency of alveolar bone between the alveolar crest and the floor of maxillary sinus is often detected in the edentulous posterior maxilla. Moreover, factors including sinus pneumatization, resorption of alveolar bone owing to tooth extraction, trauma or pathology contribute to its intensification [2,3]. This particular circumstance observed in the posterior maxilla called for a specific technique, which is sinus augmentation.

Elevation of the sinus floor was suggested to increase the bone height in the posterior area of the maxilla. First introduced by Tantum, Boyne, and James in the 1980s, this technique has been demonstrated to be highly reliable for the vertical augmentation in the maxillary posterior areas and thus has become a routine procedure [4,5]. Two kinds of approaches can be taken for the sinus augmentation, the crestal approach, and the lateral approach. The lateral approach begins with creating a lateral window to the maxillary sinus [6,7], while the crestal approach requires only a small size of osteotomy on the alveolar crest, either by using a hand or electric mallet [8,9,10]. Although various indication criteria exist, the crestal approach is not generally indicated for the residual bone height less than 4–5 mm [11,12].

Expansive adoption of CBCT has accelerated the development of digital dentistry. The most widely used radiograph in dental clinics is panoramic film, but this can cause enlargement of measures by up to 25 % [13]. Therefore, three-dimensional radiography is considered more useful in detecting the accurate widths of alveolar ridge and the maxillary sinus [14]. In addition, it also provides a detailed information on septa and sinus pathology [15]. However, CBCT imaging alone could not overcome all the challenges clinicians face during the implant surgery. Although it helped clinicians with the diagnosis and the planning for safe surgery, delivery of the surgical plan for the accurate placement of implant was still challenging. To overcome the limitation, surgical guide designed and fabricated based on the CBCT image was invented for the implant placement. The use of CBCT and surgical guide in the implant surgery has been reported to be helpful for the safe and accurate surgery [16]. Development in the digital dentistry has contributed to the astonishing advancement in implant dentistry.

When it comes to the cases requiring a simultaneous sinus augmentation for implant placement, a more advanced device than a simple surgical guide only designed for the implant placement is required. Moreover, surgical procedure can experience difficulties by severely atrophic maxilla which requires lateral approach rather than crestal approach. Considering that sinus augmentation is relatively an advanced technique for the implant dentistry, a surgical guide indicating both the path for the implant and the location of the lateral window is indispensable in the evolution of the implant dentistry. Indeed, numerous types of surgical guides for the lateral window opening have been introduced, but the massive volume of the suggested guides and the complexity of its fabrication process were major shortcomings.

The purpose of this study is to introduce a simple method for safe and precise lateral window opening for sinus augmentation using a navigation program. The study further aims to verify the effectiveness of the technique for implant placement and sinus augmentation. To the best of our knowledge, it is the first study to fabricate a sinus guide with the help of the navigation program.

## 2. Materials and Methods

### 2.1. Ethics Statement

This study was approved by the Institutional Review Board (IRB) of Hallym University Sacred Heart Hospital (IRB number: 2020-05-010) and was conducted in accordance with the Declarations of Helsinki. A written/verbal informed consent was obtained from each participant.

### 2.2. Patient Screening

**Figure 1:**
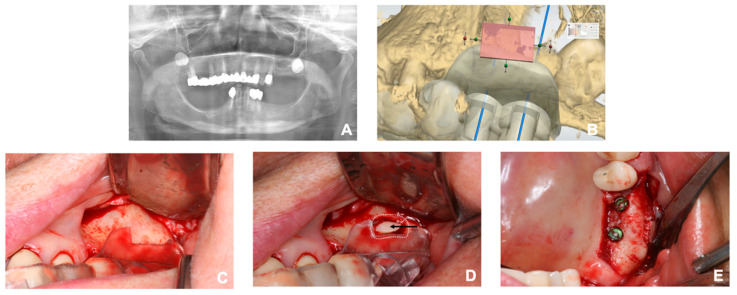
(**A**) Preoperative panoramic view of a patient. Sinus augmentation and simultaneous implant placement was planned on the left posterior region of the maxilla. (**B**) Digital planning of the surgical guide. Pink box was located to puncture out the lateral window. Note the location of the third molar. (**C**) The prefabricated surgical guide adapted to the surgical site. (**D**) A rectangular lateral window and the preserved bony plate; the white dotted line circumscribes the former and the black arrow indicates the latter. (**E**) Sinus augmentation and implant placement completed according to the surgical plan.

The patients who were qualified for the sinus augmentation with simultaneous placement were screened by using panoramic radiograph and CBCT (Figure 1A). Each criterion for the inclusion and exclusion to the study was respectively identical with the indications and the contraindications for the implant surgery and sinus augmentation. Additionally, those who had the vertical alveolar bone of 4 mm or less were considered eligible for the study. A total of five patients have participated in the study. Descriptive characteristics of each patient are organized in a table (Table 1).

### 2.3. Fabrication of the Surgical Guide

**Figure 2:**
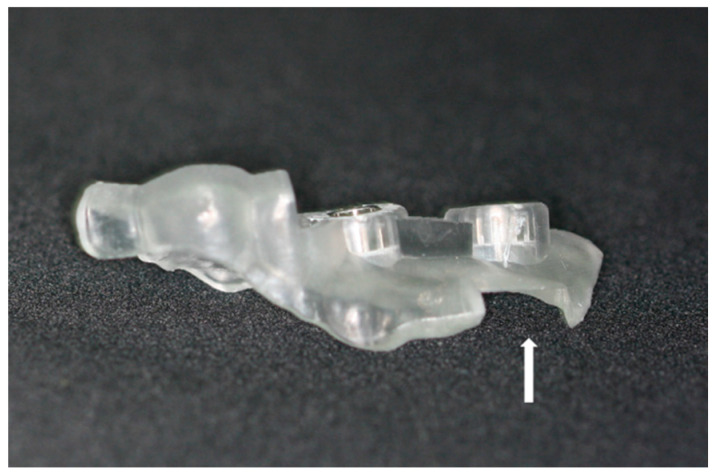
Fabricated surgical guide. The white arrow is pointing at the lateral window area.

**Figure 3:**
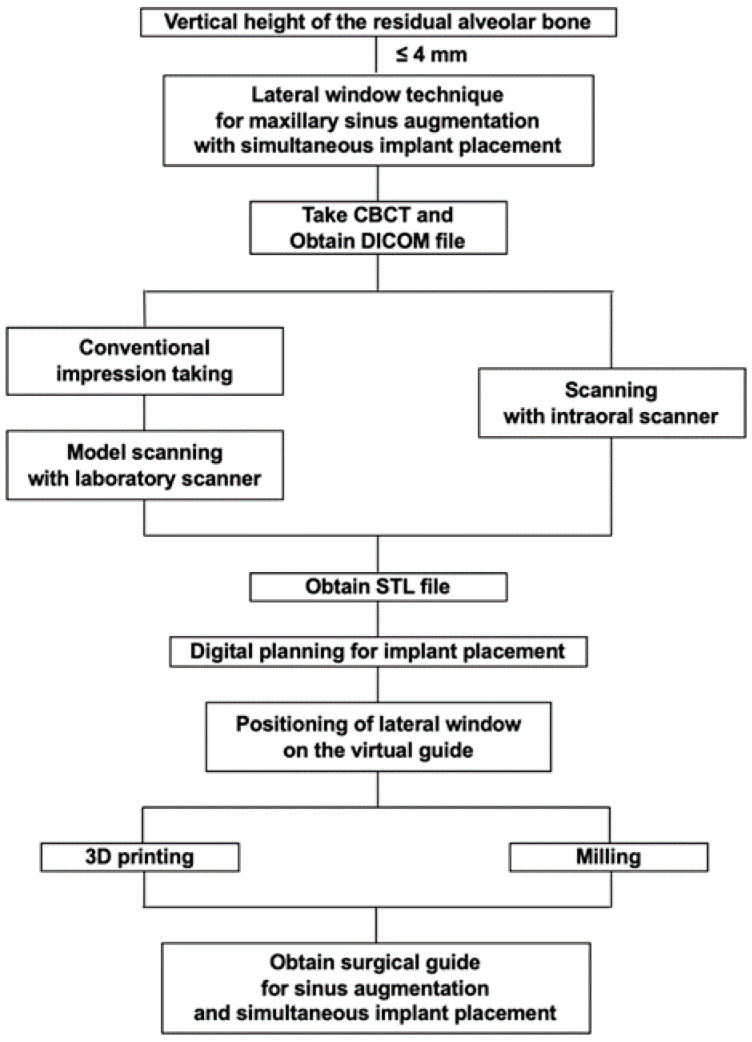
Workflow of the preparation procedure for the surgery.

Careful diagnosis and treatment planning were made based on the tomographic images. To take an accurate image of the dentition, scanning of the intraoral landscape has been completed either with intraoral scanner (Medit i500; Medit Corp., Seoul, Korea) or laboratory scanner (Rainbow Scanner, Dentium, Seoul, Korea). As for the laboratory scanner, a conventional impression was taken with irreversible hydrocolloid to make a diagnostic cast. The cast was then scanned with a laboratory scanner. The scanned data of the cast was exported as a standard tessellation language (STL) file, while the CBCT image was saved as Digital Imaging and Communications in Medicine (DICOM) data. Design of the surgical guide proceeded with superimposition of the STL file to the CBCT data through a software (3 Shape Implant studio, 3Shape A/G, Copenhagen, Denmark; Digital Guide, Dentium, Seoul, Korea). After proper adjustment, the adequate position of the implant was planned with the creation of metal or open sleeve. In the cases where there were adjacent teeth on the mesial or the distal side, at least two adjacent teeth were included to the surgical guide. In addition, the appropriate location of the lateral window was planned. (Figure 1B) Adjustment of its mesiodistal position was made with consideration on the positions of the third molar, sinus septa, and adjacent teeth or implants. The mesial and the distal boundaries of the window were set at least 1.5 mm away from the adjacent teeth or implants. The bottom of the lateral window was to be formed at least 3 mm apart from the inferior border of maxillary sinus [17]. After the location of the lateral window was determined, the inferior 3/4 of the window was punched out in a rectangular shape for the lateral window opening. The completed design of the surgical guide was then exported as an STL file. The virtual surgical guide was imported into a printing software (3D sprint, CEP Tech, Seoul, Korea) and fabricated either through milling or light polymerization via desktop printer (ProJet 6000HD, 3D Systems, Inc., Rock Hill, SC, USA) (Figure 2). The procedure prior to the surgery is briefly demonstrated as a flowchart (Figure 3). The surgical guide was finally sterilized through gamma irradiation process before the operation [18].

### 2.4. Surgical Procedure

Surgical procedure was performed by a single surgeon, starting with adaptation of the prefabricated guide to the surgical site (Figure 1C). The guide needed to be stabilized firmly without any movement. Then, the pre-planned positions of the implants were marked with a marking pen. After removing the surgical guide from the surgical site, a crestal incision was made 1 mm palatal to the marked point. The incision was further extended with sulcular incision and vertical incision starting from the line angle of the mesial tooth. The additional vertical incision was made in a lateral direction on the distal region. A full-thickness mucoperiosteal flap was reflected adequately enough to expose the lateral wall of the maxillary sinus as well as the alveolar crest. After the surgical guide was adapted again to the bone, the desired positions of the implants were marked with a surgical pencil. Cutting edges of the surgical guide were also tracked with the pencil. To form the lateral window, both the mesial and distal borders were extended superiorly and connected to each other to form a superior border. The fact that the guide tracks the inferior 3/4 of the planned window was noted during this procedure. In this way, the rectangular shaped lateral window was completed. A cut along the cutting edge without pencil marks was made when the marks were unnecessary. A lateral window was created using either a high-speed bur or a specific tool (Dentium New Sinus Kit, Dentium, Seoul, Korea). In case of the former, the bony plate was well preserved (Figure 1D). Afterwards, the sinus membrane was reflected and elevated with a surgical curette. It was handled with care to avoid iatrogenic perforation. Implant fixtures (SuperLine, Dentium, Seoul, Korea) were then placed with the identical surgical guide followed by the placement of the xenograft and alloplastic material (Geistlich Bio-Oss, Geistlich Pharma A/G, Wolhusen, Switzerland; Osteon3, Dentium, Seoul, Korea) beneath the elevated sinus membrane. In case where the bony plate has been preserved, the lateral window was covered with the plate (Figure 1E). Subsequently, the surgical site was sutured with 4–0 polyamide. All the subjects took post-operative CBCT to evaluate the outcome. The assessment of clinical complications including pain, swelling, nausea, epistaxis, or early loss of the implants was also proceeded.

## 3. Results

**Figure 4:**
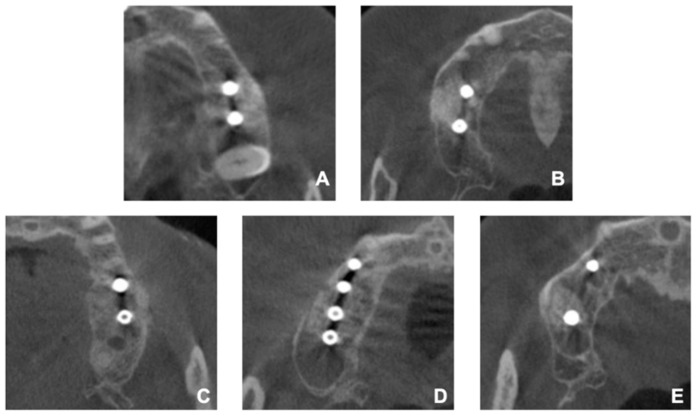
CBCT images of the patients after the operation. (**A**) Patient 1, (**B**) Patient 2, (**C**) Patient 3, (**D**) Patient 4, (**E**) Patient 5.

All five patients who underwent sinus augmentation and simultaneous implant placement with the surgical guide presented no sign of clinical complications such as pain, swelling, nausea, epistaxis, or early loss of the implants. The post-operative radiographic evaluation, performed by comparing the location of the grated site and the fixtures with the location of the lateral window based on the CBCT image in an axial view, revealed that the fixtures and the grated bone were placed accurately on the planned position. In all the patients, the fixtures were positioned within the lateral borders of the lateral window, soundly covered by the grated bone (Figure 4).

## 4. Discussion

Certain anatomic structures complicate the surgical procedure for sinus augmentation. The maxillary third molar is one of them. The impaction of the maxillary third molar is commonly found with the reported incidence of 58.87% [19]. Surgical procedure can be easier if the third molar is absent, but several complications should arise after the extraction. The oroantral communication may develop which leads to the oroantral fistula or infection of the maxillary sinus as well as the graft site if left undiagnosed or untreated [20]. Excessive removal of the bone can also occur, especially on the maxillary tuberosity [21], which has been claimed to cause hemorrhage even in the subconjunctival region [22]. Another cause that compromises the procedure is sinus septum. Septa are present in approximately 31% of patients and are found more frequently in edentulous atrophic maxilla than dentate maxilla [23]. The surgical guide fabricated based on the thorough analysis of the CBCT data allows accurate identification of the septa and leads to safer sinus augmentation.

One of the chronic problems of the conventional surgical guide has been its massive volume [24]. It requires not only sufficient intraoral space but also huge reflection of the flap. The excessive reflection of the flap is responsible to the long operation time, possibility of the nerve injury such as infraorbital nerve, post-operative swelling, and discomfort. On the contrary, the present surgical guide has greatly reduced its volume by containing only the essential information. The mesial, distal and inferior boundaries of the lateral window play a fundamental role in a rational consideration for determination of the position of the lateral window [25]. Moreover, the surgical guide also entails the estimated location of the superior margin, since the cutting edge traces the inferior half of the lateral window.

**Figure 5:**
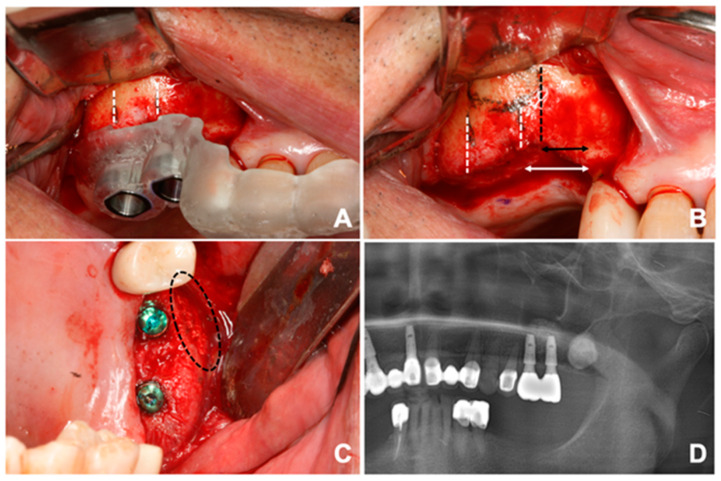
(**A**) The position of the lateral window originally planned. Each white line present mesial and proximal boundary of the window. (**B**) Black line represents the mesial border of the actual window the surgeon marked. Note the arrows to see the different distances. (**C**) The window is formed in a mesial position. (**D**) Panoramic radiograph of the restoration with prosthesis after the surgery.

In the actual surgery, a surgeon’s field of vision often varies according to his/her standing point. It is further obstructed mainly due to the mesial dentition or buccal mucosa. Therefore, the field of view tends to be distorted without a proper guide. Indeed, the surgeon suspected the accuracy of the digital planning during the first surgery using the present method (Figure 5). The lateral window on the surgical guide seemed to be located slightly to the distal side. Therefore, the guide was removed, and a new position of the lateral window was marked a little mesial to the planned site with a pencil. However, the result revealed that the surgical guide was correct, while the new position was not.

The sinus guide was first introduced by Mandelaris and Rosenfeld [26,27]. In the literature, the use of two cutting guides was proposed, contrary to the present technique using a single surgical guide. Another huge disadvantage to the suggested guide of previous studies was the complexity of procedure prior to surgery. Designing through the suggested program could be burdensome because the program was not invented primarily for the implant dentistry and required to build every single structure as well as the lateral window. The present technique differs in that the navigation system was used for simple and short planning procedures.

Ahmed has suggested computer-aided design/computer-aided manufacturing (CAD/CAM) generated surgical guide for the sinus augmentation with simultaneous placement of the implants [28]. According to his protocol, the guide was fixated in place using titanium screws for the stability. Although it succeeded in achieving immobility during the surgery, interference with the path of implant, additional bone removal from screw and demand for solid bone quality remain as its limitations. Moreover, the inconvenience in withdrawing and adapting the guide during the surgery is a major drawback since it is recommended to remove the guide as elevating the sinus floor. On the other hand, the tooth-borne surgical guide ensures favorable stability with more convenience.

Various approaches to the minimally invasive sinus augmentation have been proposed. Giovanni and colleagues have reported a high success rate of localized management of sinus floor technique in fresh molar sockets, showing a stable increase in both the vertical and the horizontal dimensions of alveolar bone [29]. While the present study focuses mainly on the atrophic maxilla with early loss of the maxillary molar(s), the localized management of sinus floor can be considered a treatment of choice in case where one of two maxillary molar extraction is indicated. On the other hand, a transcrestal approach to the sinus floor elevation has been claimed to be successful after 16 years of follow-up, even in the residual alveolar bone of less than 3 mm [30]. Although this procedure may be highly minimally invasive, it requires a skillful hand of an experienced surgeon and two steps for the implant placement.

Limitations of the study can be attributed to the small sample size. However, considering that the method introduced in this study is achieved merely by giving a modification to the existing process and that this study does not require any significant statistic result, it is believed to be acceptable. Further research with larger sample size should be made in the future.

Possible disadvantages of this technique include the lack of ability to cover from the cusps of the teeth to higher points than the buccal vestibule. The surgical guides in the previous studies could cover any range, since the designing was performed on the CBCT images. Although the surgical guide the authors present in this study is applicable without compromise in most of the cases, some cases requiring broader coverage may arise. The first situation would be with the thick residual bone, but this would not cause a problem since the crestal approach would be sufficient for the sinus augmentation. The other would be where the high alveolar ridge is seen because of severe atrophy of the maxilla. Unfortunately, this would be the case in which this new guide cannot be properly fabricated. However, the program the authors used is currently in the process to enable the design on CBCT image, not merely on the cast scanned. Future techniques may allow more stable coverage in such cases. At present, the surgical technique we introduce presents as simple but accurate method while achieving stable results as in the previous studies.

## 5. Conclusions

Lateral window opening technique combined with digital navigation system merits in simplicity and validity compared to previous methods. It is expected to be a promising technique for precise as well as safe sinus augmentation.

## Figures and Tables

**Table 1:** Patient characteristics.

Patient	Age	Gender	Implant Site	Scanning	Fabrication
Patient 1	74	F	26,27	LS	3D printing
Patient 2	68	F	16,17	LS	Milling
Patient 3	66	M	26,27	LS	Milling
Patient 4	45	M	14,15,16,17	LS	Milling
Patient 5	66	M	15,17	IS	Milling

F: female, M: male, LS: laboratory scanner, IS: intraoral scanner; Tooth numbers are identified based on Federation Dentaire International (FDI) two-digit system.
